# Prevalence and prognosis significance of cardiovascular disease in cancer patients: a population-based study

**DOI:** 10.18632/aging.102301

**Published:** 2019-09-27

**Authors:** Dong Liu, Zhiqiang Ma, Jingang Yang, Min Zhao, Huiping Ao, Xiaodong Zheng, Qianfa Wen, Yuejin Yang, Jiangyun You, Shubin Qiao, Jiansong Yuan

**Affiliations:** 1 State Key Laboratory of Cardiovascular Disease, Fuwai Hospital, National Center for Cardiovascular Diseases, Chinese Academy of Medical Sciences and Peking Union Medical College, Beijing 100037, China; 2 Department of Thoracic Surgery, Tangdu Hospital, The Fourth Military Medical University, Xi’an 710038, China; 3 Yunnan Cancer Hospital, Kunming 650221, China; 4 Jiangxi Cancer Hospital, Nanchang 330029, China; 5 Chongqing Cancer Hospital, Chongqing 400030, China; 6 Shanxi Provincial Cancer Hospital, Taiyuan 030013, China; 7 Guang’anmen Hospital of Traditional Chinese Medicine, Beijing 100053, China

**Keywords:** tumor, comorbidity, cardiovascular disease, prevalence, mortality

## Abstract

Background: Cardiovascular disease (CVD) is a heavy burden on cancer patients worldwide. This study aimed to evaluate the prevalence and influence of cardiovascular risk factors (CVRF) and CVD on the all-cause mortality among Chinese cancer patients.

Results: Overall, 13.0% of all cancer patients had at least one type of CVRFs and 5.0% with CVDs. Patients with CVRF or CVD presented more frequently at later stages and received higher percentage of oncotherapy. During 1,782,527 person-years of follow-up, the all-cause mortality in cancer patients with CVDs and with CVRFs was higher compared with those without (182.6/1000, 109.5/1000 and 93.3/1000 person-years, respectively). Cox regression analysis showed that patients with heart failure (HR 1.79, 95% CI 1.61-1.99), myocardial infarction (HR 1.50, 95% CI 1.16-1.95), atrial fibrillation (HR 1.30, 95% CI 1.09-1.53), stroke (HR 1.21, 95% CI 1.11-1.32), hypertension (HR 1.10, 95% CI 1.04-1.16) and diabetes (HR 1.16, 95% CI 1.08-1.24) had increased all-cause mortality, whereas dyslipidemia patients had better prognosis (HR 0.73, 95% CI 0.64-0.83). Stratified by cancer type, the prognostic impact of specific CVRF or CVD varied.

Methods: We consecutively recruited 710,170 cancer patients between Feb. 1995 and Jun. 2018. A stratified Cox proportional hazards model was used to analyze the effect of comorbidities on the overall survival of patients stratified by cancer type.

Conclusions: Cancer patients are vulnerable to comorbidity related to heart and cerebral disease. The influence of comorbidities on prognosis is noticeable and specific both for the type of cancer and comorbidities.

## INTRODUCTION

As the data from The Global Burden of Disease 2015 Study (GBD 2015) showed, the majority of death worldwide was caused by cardiovascular disease and cancer [1]. Due to advances in detection, early diagnosis, treatment and supportive care, cardiovascular and cancer mortality rates have declined in recent years [1, 2]. However, the increasing incidence of cardiovascular disease (CVD) in patients affected by cancer is noticeable [3–5]. Even worse, in certain groups of cancer patients, studies suggested that death from cardiovascular disease was more common than death from cancer [4, 5]. This may be the result of cardiotoxicity of cancer treatment, including chemotherapy drugs, radiotherapy and adjuvant combination therapies, which exerts adverse effects on heart function and structure [4]. Moreover, due to an overlapping of risk factors, such as smoking, obesity, air pollution and diet, patients with cancer undergo accelerated development of CVRF and CVD [6].

Recent years saw a series of guidelines about cardiovascular monitoring and clinical decision-making among cancer patients [7, 8]. Although cardio-oncology has received enormous focus, the data of cancer patients who experience CVD in China is scarce. Consequently, we collected in-patient records of a large cohort of more than seven hundred thousand cancer patients in four cancer specialty hospitals of different provinces in China. We followed the patients more than one decade and evaluated the prevalence of various CVRF and CVD in cancer patients and its impact on mortality among the general population and patients of specific cancer type, adjusting for confounders.

## RESULTS

Among 710,170 Chinese cancer patients included, 339,727 (47.8%) were male and 370,443 (52.2%) female, with a mean age of 55.1±13.1 years at cancer diagnosis and 57.4±13.2 years at follow-up (Table 1). 16.7% of patients were younger than 45 years at diagnosis, and 4.9% were younger than 35 years. The six most common types of cancer in the study were lung (18.8%), breast (24.4%), cervix uteri (20.3%), colorectal (10.0%), esophagus (8.3%) and stomach (6.8%).

**Table 1:** Baseline characteristic.

	**Total (n=710170)**	**With CVRF (n=92097, 13.0%)**	**With CVD (n=35752, 5.0%)**	**No CVD, no CVRF (n=582321, 82.0%)**	***P***
**Gender (male)**	339727 (47.8%)	42436 (46.1%)	21451 (60.0%)	275840 (47.4%)	<0.001
**Age (years)**	57.6±13.0	62.1±10.9	67.3±10.2	56.4±13.1	<0.001
**Age at diagnosis**	55.1±13.1	60.1±10.9	65.5±10.3	53.7±13.0	<0.001
**Tumor category**					<0.001
–**Lung**	133237 (18.8%)	16653 (18.1%)	10712 (18.2%)	105872 (21.4%)	
–Breast*	90193 (24.3%)	9481 (19.1%)	2308 (16.1%)	78404 (25.6%)	
–Cervix Uterus*	75037 (20.3%)	9774 (19.7%)	2100 (14.7%)	63163 (20.6%)	
–**Colon and rectum**	71202 (10.0%)	11226 (12.2%)	4390 (12.3%)	55586 (9.6%)	
–**Esophagus**	58648 (8.3%)	7543 (8.2%)	3912 (10.9%)	47193 (8.1%)	
–**Stomach**	48294 (6.8%)	5115 (5.6%)	2504 (7.0%)	40675.0 (7.0%)	
–**Thyroid**	31286 (4.4%)	4438 (4.8%)	571 (1.6%)	26277.0 (4.5%)	
–**Liver**	29884 (4.2%)	3099 (3.4%)	798 (2.2%)	25987 (4.5%)	
–**Oral, pharynx and larynx**	28654 (4.0%)	2945 (3.2%)	944 (2.6%)	24765 (4.3%)	
–**lymphoma**	23485 (3.3%)	3044 (3.3%)	1299 (3.6%)	19142 (3.3%)	
Tumor stage**	n=122543	19441 (15.9%)	11208 (9.1%)	91894 (75.0%)	<0.001
–**I**	16451 (13.4%)	2592 (13.3%)	1150 (10.3%)	12709 (13.8%)	
–**II**	25438 (20.8%)	4001 (20.6%)	1989 (17.7%)	19448 (21.2%)	
–**III**	31253 (25.5%)	4895 (25.2%)	3220 (28.7%)	23138 (25.2%)	
–**IV**	49401 (40.3%)	7953 (40.9%)	4849 (43.3%)	36599 (39.8%)	
Treatment**	n=273276	37929 (78.7%)	17339 (77.4%)	218008 (46.1%)	
–**Chemotherapy**	139217 (29.4%)	20292 (42.1%)	8720 (38.9%)	110205 (27.4%)	<0.001
–**Radiotherapy**	65400 (13.8%)	9920 (20.6%)	4669 (20.8%)	50811 (12.6%)	<0.001
–**Surgery**	240321 (50.8%)	30588 (63.5%)	14737 (65.8%)	194996 (48.5%)	<0.001

*Breast and Cervix uterus tumor were analyzed in women.

**122543 cases had records of tumor staging. 472870 cases had records of cancer related treatment. Of them, 273276 cases had received treatments.

### Cardiovascular related comorbidity prevalence

Overall, 18.0% of the cohort were affected by at least one type of cardiovascular comorbidities. In detail, 13.0% of the population had at least one type of CVRF and 5.0% had CVD. Compared with control group, patients with CVD and CVRF were older (65.5±10.3 vs 60.1±10.9 vs 53.7±13.0, *P*<0.001). In addition, patients with CVD or CVRF presented at later stages (72.0% vs 66.1% vs 65.0%, *P*<0.001) and received chemotherapy, radiotherapy and surgery more commonly (38.9% vs 42.1% vs 27.4%, 20.8% vs 20.6% vs 12.6%, 65.8% vs 63.5% vs 48.5%, *P*<0.001).

**Figure 1:**
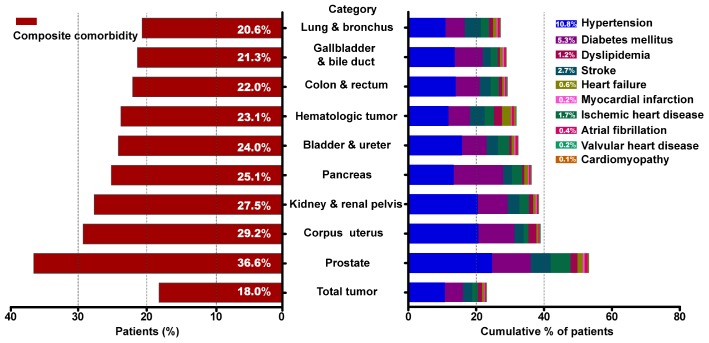
**Prevalence of cardiovascular comorbid condition by type of malignancy.** (**A**) Prevalence of cancer patients suffered from cardiovascular risk factors or cardiovascular diseases. (**B**) Cumulative percentage of cancer patients affected by individual comorbidities.

As shown in Figure 1, the most common CVRF among total cancer patients was hypertension (10.8%), followed by diabetes mellitus (5.3%) and dyslipidemia (1.2%). The highest prevalence of CVD was identified for stroke (2.7%), coronary heart disease (1.7%) and heart failure (0.6%). Patients with prostate cancer had the highest prevalence of comorbidities (36.6%), followed by patients with corpus uterus (29.2%), kidney (27.5%), pancreas (25.1%) and bladder (24.0%) cancer. The prevalence of hypertension was highest among patients with prostate cancer (24.6%) and patients with corpus uterus (20.6%). The prevalence of heart failure was highest among blood cancer patients (2.6%). More detailed information on comorbid conditions according to cancer categories was listed in Supplementary Table 1.

**Figure 2:**
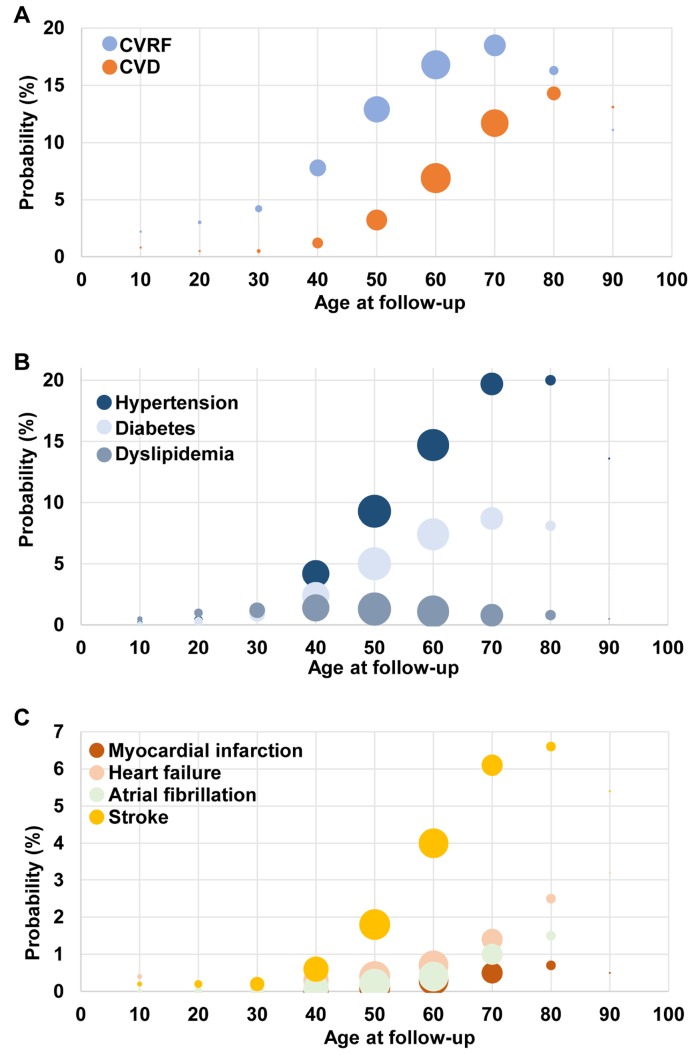
**Prevalence of cardiovascular comorbid condition by age groups.** Comparison among cancer patients (**A**) cardiovascular disease (CVD) and cardiovascular risk factor (CVRF); (**B**) CVRF; (**C**) CVD.

Cancer patients continuously and increasingly suffered from CVD with age (Figure 2). Similar trend remained in patients with heart failure and atrial fibrillation after stratification. However, the proportion of stroke, myocardial infarction and CVRF with age was reduced in elder patients after 80-year old. There were heart failure (0.4%) and stroke (0.2%) cases among childhood cancers, of which the incidence was low but noteworthy.

### Prognosis impact

**Figure 3:**
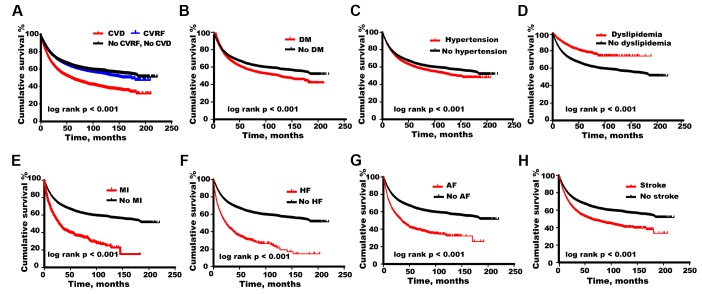
**Overall survival calculated by Kaplan–Meier curves.** Comparison of cumulative survival among cancer patients with (**A**) cardiovascular disease (CVD), cardiovascular risk factor (CVRF), (**B**) diabetes mellitus (DM), (**C**) hypertension, (**D**) dyslipidemia, (**E**) myocardial infarction (MI), (**F**) heart failure (HF), (**G**) atrial fibrillation (AF), (**H**) stroke and those without corresponding comorbidities.

During 1,782,527 person-years of follow-up (median follow-up time 2.5 years), 174,482 patients (24.6%) died with all-cause mortality rate of 97.9 (95% CI 97.4-98.3) per 1000 person-years. Cancer patients with either CVD or CVRF had higher mortality of 182.6 (95% CI 179.5-185.7) and 109.5 (95% CI 108.1-111.0) per 1000 person-years respectively compared with control group [93.3 (95% CI 92.9-93.8) per 1000 person-years] (Figure 3A). The age-standardized mortality rate by world Segi’s standard population was 494.5/1000 in CVD patients, 84.4/1000 person-years in CVRF patients, 102.9/1000 person-years in cancer patients without heart comorbidity and 105.7/1000 person-years in the whole cohort respectively.

However, results varied by CVD categories (Figure 3B–3H). For hypertension, diabetes mellitus, myocardial infarction, heart failure, atrial fibrillation or stroke, patients with corresponding comorbidities had lower survival probabilities (*P* < 0.001). Interestingly, all-cause mortality in cancer patients with dyslipidemia was relatively lower (*P* < 0.001).

After adjustment for age, sex, tumor stage and treatment, multivariable regression analysis (Table 2) presented that diabetes mellitus (HR 1.15, 95% CI 1.08-1.24, *P*<0.001), myocardial infarction (HR 1.50, 95% CI 1.16-1.95, *P*=0.002), heart failure (HR 1.79, 95% CI 1.61-1.99, *P*<0.001), atrial fibrillation (HR 1.30, 95% CI 1.09-1.53, *P*=0.003) and stroke (HR 1.21, 95% CI 1.11-1.32, *P*<0.001) were independent risk factors for all-cause mortality, while dyslipidemia remained to be a protective factor for survival (HR 0.73, 95% CI 0.64-0.83, *P*<0.001) in cancer patients.

**Table 2:** Factors associated with all-cause mortality in tumor patients.

	**P**	**HR**	**95%CI**
**Female**	<0.001	0.692	0.666	0.719
**Age**	<0.001	1.009	1.008	1.011
**Surgery**	<0.001	0.734	0.706	0.763
**Chemotherapy**	<0.001	0.913	0.877	0.951
**Radiation**	<0.001	0.788	0.749	0.828
**Tumor stage**	<0.001	3.105	2.944	3.275
**Hypertension**	0.002	1.095	1.035	1.159
**Diabetes**	<0.001	1.153	1.077	1.235
**Dyslipidemia**	<0.001	0.728	0.641	0.826
**Myocardial infarction**	0.002	1.502	1.155	1.953
**Heart Failure**	<0.001	1.787	1.607	1.988
**Atrial Fibrillation**	0.003	1.296	1.094	1.534
**Stroke**	<0.001	1.207	1.109	1.315

**Figure 4:**
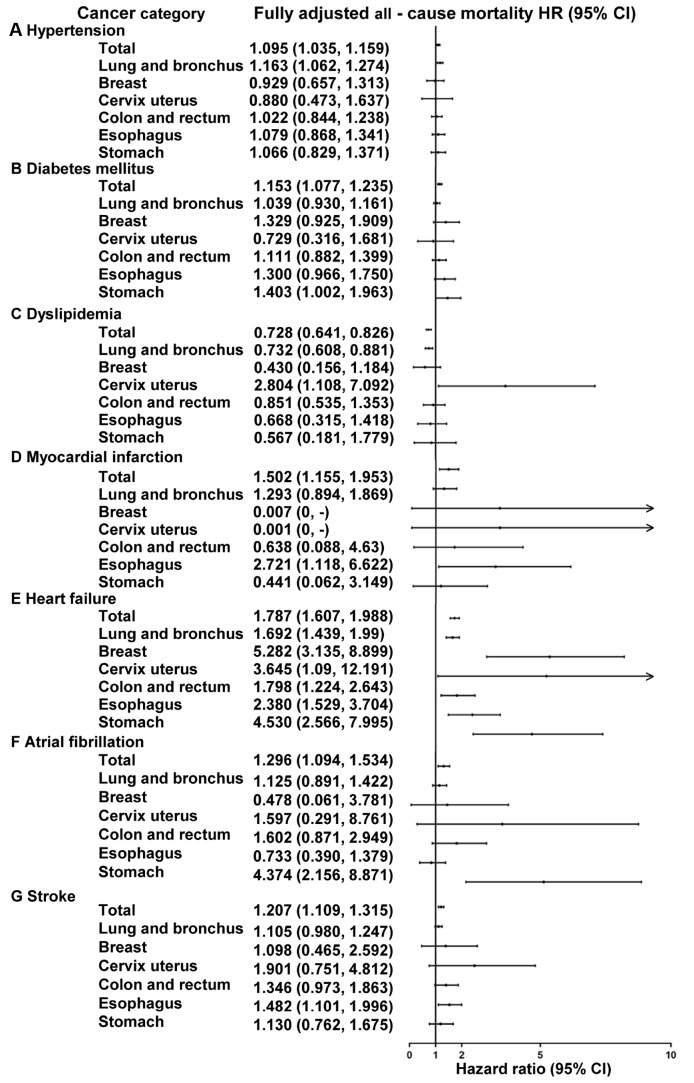
**Hazard ratios for all-cause mortality in top six cancer types patients with and without specific cardiovascular comorbidities.** The impact of the specific CVRF or CVD on mortality for the general cancer patients, lung and bronchus, breast, cervix uterus, colon and rectum, esophagus and stomach cancer patients. Hazard ratios from Cox regression analysis were adjusted for age, gender, treatment, tumor stage and cardiovascular comorbidities.

We also evaluated the impact of CVRF and CVD on the all-cause mortality stratified by cancer categories (Figure 4 and Supplementary Table 2). Adjustment for age and gender resulted in minor changes, whereas after taking tumor stage, treatment and other CVD comorbidity into account, all hazard ratios changed significantly. After fully adjusted among the six most prevalent malignancies (Figure 4), lung cancer patients with hypertension still had poorer prognosis (HR1.16, 95% CI 1.06-1.27) and with dyslipidemia had lower all-cause mortality (HR 0.73, 95% CI 0.61-0.88). However, in cervical cancer patients, dyslipidemia was associated with higher all-cause mortality (HR 2.80, 95% CI 1.11-7.09). The association between heart failure and mortality was strong in lung (HR 1.69, 95% CI 1.44-1.99), breast (HR 5.27, 95% CI 3.14-8.90), cervix uteri (HR 3.65, 95% CI 1.09-12.19), colorectal (HR 1.80, 95% CI 1.22-2.64), esophagus (HR 2.38, 95% CI 1.53-3.70) and stomach cancer (HR 4.53, 95% CI 2.57-8.00). Esophagus cancer patients with myocardial infarction or stroke had higher rates of mortality (HR 2.72, 95% CI 1.12-6.62; HR 1.48, 95% CI 1.10-2.00). A diagnosis of either diabetes mellitus or atrial fibrillation in stomach cancer patients was independently associated with decreased survival probabilities (HR 1.40, 95% CI 1.00-1.96; HR 4.37, 95% CI 2.16-8.87, respectively).

### Treatment associated risk in heart failure patients

Since the HR of all-cause mortality was significantly increased for heart failure, we evaluated the association between cancer related treatment and mortality in lung, breast, esophagus and stomach cancer patients with heart failure, whose radiotherapy targets may cover heart regions (Table 3). For lung cancer patients with heart failure, chemotherapy (HR 0.58, 95% CI 0.39-0.86) and chemo-radiotherapy (HR 0.38, 95% CI 0.25-0.59) was still associated with a lower mortality, whereas the effect of radiotherapy was uncertain. The conclusion was similar among all cancer patients combined but not each type of cancer.

**Table 3:** Association between treatment and mortality in tumor patients with heart failure*.

	**Lung HR (95% CI)**	**Breast HR (95% CI)**	**Esophagus HR (95% CI)**	**Stomach HR (95% CI)**	**Total HR (95% CI)**
**None**	Ref.	Ref.	Ref.	Ref.	Ref.
**Chemotherapy**	0.578 (0.389, 0.858)	1.206 (0.175, 8.31)	1.311 (0.337, 5.103)	0.341 (0.05, 2.328)	0.513 (0.398, 0.66)
**Radiotherapy**	0.572 (0.316, 1.033)	330759.846 (0, 1.604E+255)	1.596 (0.491, 5.188)	NA^**^	1.012 (0.711, 1.441)
**Chemotherapy + Radiotherapy**	0.384 (0.25, 0.589)	5.931 (0.868, 40.511)	NA^**^	13.927 (0.053, 3668.627)	0.489 (0.354, 0.675)

*The model of multivariate logistic regression analysis included age, gender, treatment, tumor stage, CVRF and other CVD comorbidities.

^**^ The sample is small and the HR can’t be calculated.

## DISCUSSION

**Figure 5:**
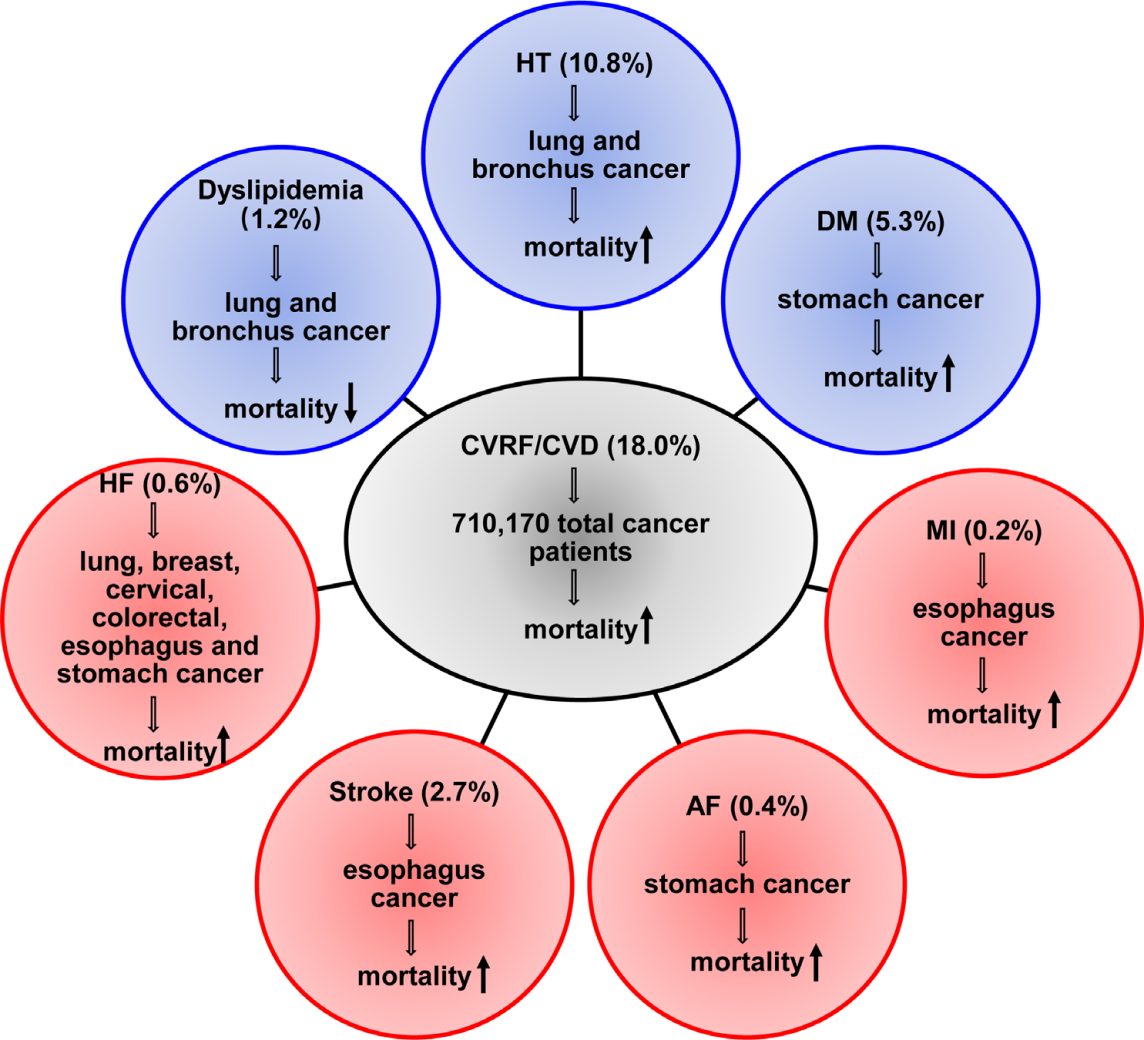
**Summary of the impact of the specific CVRF or CVD on mortality for the general cancer patients and the top six cancer types patients.** HT, hypertension; DM, diabetes mellitus; MI, myocardial infarction; HF, heart failure; AF, atrial fibrillation.

To our knowledge, this is the largest cohort to investigate CVRF and CVD comorbidities in Chinese cancer patients. Most of the previous work that has showed the prognostic impact of comorbidities on cancer was from single center without stratification by cancer type or comorbidity type limited by small sample size [9]. We extracted more comprehensive cardiovascular related diagnosis from the medical records of 710,170 candidates from multicenter. Furthermore, the present study is the first to analyze the prevalence and long-term prognosis impact of various types of cardiovascular comorbidity on cancer patients specifically. There was difference in the prevalence of selected CVD by cancer type. Most of CVRF or CVD led to higher mortality among cancer patients with the exception of dyslipidemia. The prognostic impact of specific CVD varied after stratified by cancer type (Figure 5). Finally, we focused on cancer patients with heart failure and observed that for lung cancer patients with heart failure, chemotherapy and chemo-radiotherapy improved prognosis but radiotherapy didn’t.

The prevalence of CVRF and CVD in this cohort was evidently higher than that reported in general population [1, 2]. Since the comorbidity definition and the cancer type varies, there is no consistence of the prevalence [9]. In the annual report of cancer status in the United States, 40.2% of all cancers had at least one multi-systemic comorbidity [3]. Lee *et al.* reviewed that the prevalence of comorbidity ranged from 0.4% to 90% among cancer patients [10]. The relatively low prevalence of comorbidity (18%) in our study may be related to the younger mean age of 55 years at diagnosis. Moreover, we only focused on CVD comorbidity in present study.

In our study, cancer patients with CVD comorbidities were older at diagnosis and at a more advanced stage than those without comorbidities. Grann *et al.* reported melanoma patients with comorbidity was associated with later cancer stage [11]. However, Ahn and colleagues found that lung cancer patients with comorbidities were more likely to present with early stage [12]. This impact of comorbidity on diagnosis stage may be related to the cancer types and the severity of comorbidities. Stable comorbidities may increase the frequency of physician visits and screening leading to early diagnosis, while severe ones may distract attention from detecting cancer and result in delayed cancer diagnosis.

The risk of CVD differed in various cancer types. Cancer patients of prostate, corpus uterus, kidney, pancreas, bladder, blood, colorectal, gallbladder and lung suffered from a higher prevalence of CVD in this study. However, Edwards *et al.* reported that patients with prostate cancer had similar prevalence of comorbidity as general population and lung cancer patients had more prevalence of comorbidity [3]. The inconsistence may be explained by the elder age of prostate cancer patients with an average age of 73.1 in our study. Since the incidence of heart disease and stroke increases with age significantly, the number of cancer patients with comorbidity will increase evidently. Prostate cancer patients usually carry relatively favorable prognosis and live longer. What’s worse, a large proportion of prostate cancer patients possibly experience androgen deprivation therapy (ADT) after cancer diagnosis. Hence, they are more likely to have CVD comorbidity [13].

As for the prevalence of specific comorbidity, we found hypertension was the most common comorbidity, especially in prostate and urinary bladder cancer patients. In a meta-analysis reviewed 3200 prostate cancer patients, the frequency of hypertension was more than 70% [14]. Further studies are necessary to confirm whether specific cancer and hypertension share a common mechanism.

The prevalence of diabetes was also significantly high. The high incidence of diabetes may be related to the wide use of chemotherapy in cancer treatment, cellular proliferation and development of insulin resistance. In addition, destruction of pancreatic cell by cancer and treatment results in the highest risk of diabetes mellitus in pancreatic cancer [15, 16].

We observed heart failure was relatively more common in hematologic tumors, consistent with the report of Japanese [9]. Anemia, hyper-viscosity, renal dysfunction, amyloidosis, and treatment related cardiovascular toxicities among blood cancer patients may increase the risk of heart failure [17].

Our study also found a positive association between atrial fibrillation and cancer, consistent with the results of several studies [18]. This association is not limited by surgery or medical therapy. Cancer patients without cancer-related treatments still had an increasing likelihood of harboring atrial fibrillation [19]. The inverse relationship also exists. The risk of any type of cancer is particularly high within 3 months and remained increased following the diagnosis of AF [20]. It is reasonable to hypothesize that chronic inflammation, common risk factors and autonomic dysregulation is related to the occurrence of atrial fibrillation [18].

Most of studies demonstrated comorbidity had a negative effect on survival [3, 21]. In our study, diabetes, hypertension, myocardial infarction, heart failure, atrial fibrillation and stroke increased mortality generally. The impact of serious comorbidity tends to be greater, such as myocardial infarction and heart failure. Notably, various cancer types are differently influenced by specific comorbid condition. For both breast and colorectal cancer, heart failure-but not other selected comorbidities-independently decreased survival once confounders were adjusted.

For lung cancer, hypertension is independently associated with mortality. In a cohort of 101776 lung cancer, hypertension significantly increased mortality risk in one (47.9%), five (30.5%), and ten (28.2%) years compared with other non-pulmonary comorbidities [22]. However, another study presented that non-small cell lung cancer who developed hypertension after bevacizumab therapy had improved prognosis [23].

Interestingly, we found dyslipidemia possibly had a positive effect on cancer survival, especially in lung cancer. Similarly, other Chinese investigators found lower total cholesterol (TC) level was an independent risk factor for mortality in non-small cell lung cancers patients [24]. In another study of cancer types combined, high TC, high density lipoprotein-cholesterol, apo(a), and Lp(a) levels were showed to be associated with an increase in total cancer survival [25]. A meta-analysis of 26 studies showed that higher TC before diagnosis was associated with a reduced risk of overall survival and disease-free survival [26]. More studies are needed to validate the impact of various lipid biomarkers on cancer mortality.

For atrial fibrillation, out results presented that it had adverse impact for all cancer patients combined. After adjustment for potential co-variates, the association remained in stomach cancer patients. Several studies likewise demonstrated that cancer patients with atrial fibrillation had poorer survival in non-Hodgkin lymphoma, lung cancer, colon cancer and esophageal cancer [19], while in a population-based study atrial fibrillation seemed not to increase overall mortality [27].

Esophagus cancer patients with myocardial infarction or stroke had increased overall mortality, in line with some other studies [28, 29]. In a Swedish cohort study [28] of patients after esophageal cancer surgery, investigators presented patients with squamous cell carcinoma who had stroke had a worse prognosis, whereas those with adenocarcinoma did not.

Heart failure is relatively common in cancer patients particularly after cancer treatment [4]. Considering heart failure as an evidently adverse predictor of overall survival, we subsequently analyzed the impact of treatment on heart failure. After full adjustment of age, sex, tumor stage and other CVD comorbidities, the addition of chemotherapy or chemoradiotherapy on heart failure patients remained to be associated with better prognosis in this study beyond our expectation. In a study of early-stage breast cancer in Denmark with a median follow-up of 14 years, the impact of treatment on breast cancer mortality was similar among patients with and without comorbidity [30]. Hence, the investigators suggested to adhere to the standardized treatment. Reversely, a study of 1378 prostate patients with a history of heart failure showed patients may be harmed by the addition of androgen deprivation therapy (ADT) to radiation [31]. More large-sample studies to confirm this conclusion are needed.

Although cancer registries do not routinely collect information on comorbidities and cardiovascular disease studies commonly exclude cancer patients, CVD comorbidity is increasingly common and highly relevant to the prognosis of cancer patients. Evaluation of comorbid condition is of great significance to help to deepen our understanding of cancers and their relations to other diseases. Moreover, after the cancer-related treatment, close monitoring and optimal cardiac-specific treatment was associated with preventing the CVD progress and promoting cardiac functional recovery [7]. In a long-term clinical trial, more than 80% of patients with anthracycline induced cardiotoxicity recovered after prompt heart failure therapy [32]. Additionally, timely cancer drug alteration or interruption as necessary increases the likelihood of CVD recovery [33, 34].

Clinical studies with large population and high quality are necessary to acquire valid evidence of management of CVD in cancer patients. If we could prevent, diagnose and treat comorbidity individually and properly, the heavy burden of heart disease on cancer patients would largely lessen.

## MATERIALS AND METHODS

### Study population and data collection

Data present in the current study were collected through a computer assisted personal interview system from February 1995 to June 2018, which initially collects information on only newly diagnosed primary cancer inpatients provided by 4 cancer specialty hospitals in China. Data of birthday, gender, diagnosis, tumor stage, diagnosis dates and treatment were abstracted from hospital discharge records of the participants. Each record had information on up to 30 diagnoses, which were used to identify patients’ comorbidity. The CVD diagnosis was validated by cardiologists. Follow-up information was collected by clinic visit, hospitalization or telephone call. The study was in accordance with the Institutional Review Board of the participating hospitals and waived the requirement for informed consent because we used only deidentified data.

There were 725,275 patients in the whole database. Patients who were older than 100 years old (n=10), confirmed with benign tumors or tumor-like diseases by pathologic examination (n=15,095) were excluded, resulting in 710,170 patients eligible for analyzing the CVRF and CVD comorbidity. Tumor stage was recorded in 122,543 patients and type of treatment was recorded in 472,870 patients. We excluded patients who had incomplete data of tumor stage and treatment, leaving a final sample of 62,549 cancer patients for Cox multivariate regression analysis of the association between CVRF and CVD comorbidity and all-cause mortality. The Institutional Review Board of the participating hospitals approved this study and waived the requirement for informed consent because we used only deidentified data.

CVD comorbidity was defined as a composition of heart failure, atrial fibrillation, myocardial infarction, ischemic heart disease, cardiomyopathy, valvular heart disease and stroke. CVRF comprised hypertension, diabetes and dyslipidemia. Those without CVRF or CVD were assigned as control group. The study endpoint was all-cause mortality.

### Statistical analysis

Continuous variables were presented as mean ± standard deviation (SD) and categorical variables were expressed as frequency (%). The continuous variables were compared with an analysis of variance (ANOVA) and categorical variables among groups were compared with chi square test. Survival analysis was demonstrated by Kaplan-Meier curves and the curves were compared with the log-rank test. The Segi’s World Standard population was applied for age-standardized mortality calculation. Univariable (unadjusted) and multivariable adjusted Cox proportional hazard models were conducted to evaluate the risk factors associated with mortality. Hazards ratios (HRs) for mortality and corresponding 95% confidence interval (95% CI) were calculated. The following variables were entered into the fully adjusted model: age at follow-up, gender, cardiovascular comorbidities (expect the aimed exposure), neoplasm stage and treatment. The stratified analysis was conducted to measure the impact of specific CVRF and CVD on mortality in patients with different cancer sites. In addition, the Cox regression proportional model was also applied in the investigation of the association between treatment methods and mortality in patients with heart failure with stratification of cancer sites. For all analysis reported, *P* values were 2-sided, and *P* values <0.05 were considered to be statistically significant. The statistical analyses were conducted with the use of SPSS statistical software (version 22; SPSS Inc., Chicago, IL).

### LIMITATION

Despite of the large sample size of this study, several limitations of this study also need to be considered. First, we had no data of specific causes of death. However, in a real world it is relatively difficult to figure out cancer-related death among patients with end-stage tumor and comorbidity. Second, the database does not record potential confounding factors such as obesity, tobacco consumption, family history and so on. Thirdly, the time of comorbidity emerging was not recorded. We are not exactly sure whether comorbidity occurred before cancer diagnosis or not. Based on this situation, the causal relationship between cancer and heart disease is not the focus of the present study, but the point is prevalence of the selected comorbidity and its impact on mortality. Fourthly, since the time interval is pretty long during the current study, the therapy is likely to change over time, which may affect survival analysis.

## CONCLUSION

This large-scale cohort study confirmed that cancer patients had a substantially heavy burden of CVRF and CVD. Most of cardiovascular related comorbidity exerted adverse impact on all cancer mortality, whereas hypercholesterolemia indicated a better prognosis. Prevalence of CVRF and CVD comorbidity and its impact on survival is specific for cancer type. Medical practitioners should improve medical collaboration, provide routine cardiovascular monitoring and appropriately control cardiovascular risk factors case-by-case among these vulnerable patient population.

## Supplementary Material

Supplementary Table 1

Supplementary Table 2
