# Supplementary material for: Prevalence and prognosis significance of cardiovascular disease in cancer patients: a population-based study
Source: Aging (Albany NY). 2019 Sep 27;11(18):7948–60. doi: 10.18632/aging.102301 (PMC6781987; doi:10.18632/aging.102301)
Supplement: Supplementary Table 1 [file aging-11-102301-s002.docx]

**Supplementary Table 1. Prevalence of cardiovascular comorbidities for each type of cancer.**

|  | **Cardio-vascular comorbidity** | | **DM** | **Hypertension** | **Dyslipidemia** | | **Myocardial infarction** | | **Heart failure** | | | **Atrial fibrillation** | **Stroke** | **Arrythmia** | | **Coronary heart disease** | **Cardiomyo-pathy** | | **Valvular heart disease** | |
| --- | --- | --- | --- | --- | --- | --- | --- | --- | --- | --- | --- | --- | --- | --- | --- | --- | --- | --- | --- | --- |
| Total cancers (n=710170) | 127849  (18.00 %) | 37846  (5.33%) | | 76336  (10.75%) | | 8334  (1.17%) | | 1497  (0.21%) | | 4343  (0.61%) | 2508  (0.35%) | | 19436  (2.74%) | | 13666  (1.92%) | 12039  (1.70%) | | 846  (0.12%) | 1210  (0.17%) |  |
| Lung & bronchus  (n=133237,18.76%) | 27365 (20.54%) | 7582  (5.69%) | | 14573  (10.94%) | | 1553  (1.17%) | | 458  (0.34%) | | 1395  (1.05%) | 835  (0.63%) | | 6327  (4.75%) | | 3284  (2.46%) | 3182  (2.39%) | | 185  (0.14%) | 243  (0.18%) |  |
| Breast  (n=90193,24.35%) | 11789 (13.07%) | 3958  (4.39%) | | 7471  (8.28%) | | 921  (1.02%) | | 44  (0.05%) | | 253  (0.28%) | 106  (0.12%) | | 1174  (1.30%) | | 840  (0.93%) | 867  (0.96%) | | 118  (0.13%) | 110  (0.12%) |  |
| Cervix uteri  (n=75037,20.26%) | 11874 (15.82%) | 3154  (4.20%) | | 7378  (9.83%) | | 979  (1.30%) | | 40  (0.05%) | | 265  (0.35%) | 108  (0.14%) | | 1096  (1.46%) | | 1681  (2.24%) | 576  (0.77%) | | 41  (0.05%) | 151  (0.20%) |  |
| Colon & rectum  (n=71202,10.03%) | 15616 (21.93%) | 5103  (7.17%) | | 9907  (13.91%) | | 719  (1.01%) | | 208  (0.29%) | | 374  (0.53%) | 301  (0.42%) | | 2241  (3.15%) | | 1582  (2.22%) | 1734  (2.44%) | | 88  (0.12%) | 176  (0.25%) |  |
| Esophagus  (n=58648,8.26%) | 11455 (19.53%) | 2535  (4.32%) | | 6980  (11.90%) | | 193  (0.33%) | | 196  (0.33%) | | 361  (0.62%) | 318  (0.54%) | | 2241  (3.82%) | | 1382  (2.36%) | 1225  (2.09%) | | 50  (0.09%) | 97  (0.17%) |  |
| Stomach  (n=48294,6.80%) | 7619 (15.78%) | 2049  (4.24%) | | 4515  (9.35%) | | 151  (0.31%) | | 149  (0.31%) | | 228  (0.47%) | 173  (0.36%) | | 1380  (2.86%) | | 822  (1.70%) | 830  (1.72%) | | 30  (0.06%) | 92  (0.19%) |  |
| Thyroid  (n=31286,4.41%) | 5009 (16.01%) | 1138  (3.64%) | | 3269  (10.45%) | | 877  (2.80%) | | 14  (0.04%) | | 30  (0.10%) | 25  (0.08%) | | 321  (1.03%) | | 411  (1.31%) | 203  (0.65%) | | 11  (0.04%) | 22  (0.07%) |  |
| Liver  (n=29884,4.21%) | 3897 (13.04%) | 1736  (5.81%) | | 2300  (7.70%) | | 78  (0.26%) | | 39  (0.13%) | | 136  (0.46%) | 71  (0.24%) | | 344  (1.15%) | | 238  (0.80%) | 325  (1.09%) | | 21  (0.07%) | 28  (0.09%) |  |
| Oral, pharynx & larynx  (n=28654,4.03%) | 3889 (13.57%) | 1142  (3.99%) | | 2188  (7.64%) | | 327  (1.14%) | | 39  (0.14%) | | 77  (0.27%) | 66  (0.23%) | | 543  (1.90%) | | 444  (1.55%) | 294  (1.03%) | | 28  (0.10%) | 32  (0.11%) |  |
| Lymphoma  (n=23485,3.31%) | 4343 (18.49%) | 1338  (5.70%) | | 2012  (8.57%) | | 759  (3.23%) | | 44  (0.19%) | | 395  (1.68%) | 97  (0.41%) | | 608  (2.59%) | | 589  (2.51%) | 548  (2.33%) | | 132  (0.56%) | 46  (0.20%) |  |
| Ovary  (n=18835,5.08%) | 3615 (19.19%) | 1019  (5.41%) | | 2049  (10.88%) | | 486  (2.58%) | | 20  (0.11%) | | 108  (0.57%) | 23  (0.12%) | | 315  (1.67%) | | 583  (3.10%) | 202  (1.07%) | | 11  (0.06%) | 42  (0.22%) |  |
| Other sites (n=14347,2.02%) | 2306 (16.07%) | 648  (4.52%) | | 1411  (9.83%) | | 174  (1.21%) | | 20  (0.14%) | | 58  (0.40%) | 37  (0.26%) | | 302  (2.10%) | | 292  (2.04%) | 152  (1.06%) | | 14  (0.10%) | 24  (0.17%) |  |
| Corpus uterus  (n=11270,3.04%) | 3293 (29.22%) | 1199  (10.64%) | | 2324  (20.62%) | | 261  (2.32%) | | 12  (0.11%) | | 59  (0.52%) | 34  (0.30%) | | 305  (2.71%) | | 318  (2.82%) | 159  (1.41%) | | 13  (0.12%) | 30  (0.27%) |  |
| Bladder & ureter  (n=11032,1.55%) | 2647 (23.99%) | 803  (7.28%) | | 1740  (15.77%) | | 71  (0.64%) | | 43  (0.39%) | | 85  (0.77%) | 70  (0.63%) | | 372  (3.37%) | | 221  (2.00%) | 370  (3.35%) | | 12  (0.11%) | 19  (0.17%) |  |
| Kidney &  renal pelvis  (n=8777,1.24%) | 2415 (27.52%) | 772  (8.80%) | | 1793  (20.43%) | | 115  (1.31%) | | 32  (0.36%) | | 62  (0.71%) | 28  (0.32%) | | 307  (3.50%) | | 97  (1.11%) | 243  (2.77%) | | 16  (0.18%) | 11  (0.13%) |  |
| Skin  (n=8562,1.21%) | 1452 (16.96%) | 379  (4.43%) | | 975  (11.39%) | | 144  (1.68%) | | 11  (0.13%) | | 29  (0.34%) | 34  (0.40%) | | 168  (1.96%) | | 112  (1.31%) | 141  (1.65%) | | 14  (0.16%) | 11  (0.13%) |  |
| Pancreas  (n=6652,0.94%) | 1672 (25.14%) | 975  (14.66%) | | 885  (13.30%) | | 39  (0.59%) | | 22  (0.33%) | | 72  (1.08%) | 32  (0.48%) | | 166  (2.50%) | | 72  (1.08%) | 206  (3.10%) | | 9  (0.14%) | 12  (0.18%) |  |
| Nervous system  (n=6481,0.91%) | 867 (13.38%) | 197  (3.04%) | | 455  (7.02%) | | 79  (1.22%) | | 10  (0.15%) | | 21  (0.32%) | 5  (0.08%) | | 256  (3.95%) | | 51  (0.79%) | 40  (0.62%) | | 3  (0.05%) | 6  (0.09%) |  |
| Gallbladder &  bile duct  (n=5545,0.78%) | 1179 (21.26%) | 457  (8.24%) | | 757  (13.65%) | | 36  (0.65%) | | 25  (0.45%) | | 42  (0.76%) | 30  (0.54%) | | 130  (2.34%) | | 79  (1.42%) | 118  (2.13%) | | 5  (0.09%) | 8  (0.14%) |  |
| Prostate  (n=5380,1.58%) | 1966 (36.54%) | 618  (11.49%) | | 1324  (24.61%) | | 107  (1.99%) | | 28  (0.52%) | | 87  (1.62%) | 46  (0.86%) | | 313  (5.82%) | | 158  (2.94%) | 317  (5.89%) | | 14  (0.26%) | 11  (0.20%) |  |
| Uncertain metastatic tumor (n=5028,0.71%) | 620 (12.33%) | 188  (3.74%) | | 360  (7.16%) | | 36  (0.72%) | | 9  (0.18%) | | 23  (0.46%) | 10  (0.20%) | | 120  (2.39%) | | 47  (0.93%) | 34  (0.68%) | | 4  (0.08%) | 7  (0.14%) |  |
| Other genital organs (n=4015,0.57%) | 531 (13.23%) | 166  (4.13%) | | 325  (8.09%) | | 36  (0.90%) | | 5  (0.12%) | | 9  (0.22%) | 2  (0.05%) | | 52  (1.30%) | | 59  (1.47%) | 29  (0.72%) | | 0  (0.00%) | 5  (0.12%) |  |
| Other hematologic tumor  (n=3969,0.56%) | 918 (23.13%) | 253  (6.37%) | | 468  (11.79%) | | 97  (2.44%) | | 9  (0.23%) | | 104  (2.62%) | 24  (0.60%) | | 172  (4.33%) | | 94  (2.37%) | 107  (2.70%) | | 20  (0.50%) | 12  (0.30%) |  |
| Bone  (n=3278,0.46%) | 327 (9.98%) | 101  (3.08%) | | 167  (5.09%) | | 13  (0.40%) | | 5  (0.15%) | | 16  (0.49%) | 6  (0.18%) | | 25  (0.76%) | | 75  (2.29%) | 28  (0.85%) | | 1  (0.03%) | 3  (0.09%) |  |
| Other GI organs & peritoneum (n=3113,0.44%) | 540 (17.35%) | 144  (4.63%) | | 364  (11.69%) | | 31  (1.00%) | | 9  (0.29%) | | 19  (0.61%) | 9  (0.29%) | | 70  (2.25%) | | 44  (1.41%) | 54  (1.73%) | | 5  (0.16%) | 8  (0.26%) |  |
| Other intrathoracic (n=2931,0.41%) | 487 (16.62%) | 142  (4.84%) | | 247  (8.43%) | | 42  (1.43%) | | 3  (0.10%) | | 29  (0.99%) | 14  (0.48%) | | 76  (2.59%) | | 75  (2.56%) | 42  (1.43%) | | 1  (0.03%) | 4  (0.14%) |  |
| Connective issue  (n=592,0.08%) | 81 (13.68%) | 23  (3.89%) | | 56  (9.46%) | | 4  (0.68%) | | 1  (0.17%) | | 3  (0.51%) | 2  (0.34%) | | 5  (0.84%) | | 7  (1.18%) | 9  (1.52%) | | 0  (0.00%) | 0  (0.00%) |  |
| Mesothelioma  (n=324,0.05%) | 64 (19.75%) | 24  (7.41%) | | 33  (10.19%) | | 3  (0.93%) | | 2  (0.62%) | | 3  (0.93%) | 2  (0.62%) | | 7  (2.16%) | | 7  (2.16%) | 4  (1.23%) | | 0  (0.00%) | 0  (0.00%) |  |
| Other pelvic cavity  (n=119,0.02%) | 13 (10.92%) | 3  (2.52%) | | 10  (8.40%) | | 3  (2.52%) | | 0  (0.00%) | | 0  (0.00%) | 0  (0.00%) | | 0  (0.00%) | | 2  (1.68%) | 0  (0.00%) | | 0  (0.00%) | 0  (0.00%) |  |

Abbreviations: DM, diabetes mellitus; GI, gastrointestinal.
