# Supplementary material for: Prevalence and prognosis significance of cardiovascular disease in cancer patients: a population-based study
Source: Aging (Albany NY). 2019 Sep 27;11(18):7948–60. doi: 10.18632/aging.102301 (PMC6781987; doi:10.18632/aging.102301)
Supplement: Supplementary Table 2 [file aging-11-102301-s001.docx]

**Supplementary Table 2. Association between specific heart disease and all - cause mortality in cancer patients stratified by cancer site**s.

|  | **Lung & bronchus** | **Breast** | **Cervix uterus** | **Colon & rectum** | | **Esophagus** | **Stomach** | | **Thyroid** | | **Liver** | | **Oral,**  **pharynx &larynx** | | | **Lymphoma** |
| --- | --- | --- | --- | --- | --- | --- | --- | --- | --- | --- | --- | --- | --- | --- | --- | --- |
| **Diabetes Mellitus** |  |  |  |  |  |  |  |  |  |  |  |  |  |  |  |  |
| Unadjusted  HR (95% CI) | 1.12 (1.01,1.25) | 1.45 (1.03,2.04) | 0.79 (0.37,1.70) | 1.22 (0.98,1.51) | 1.36 (1.01,1.81) | | | 1.49 (1.08,2.05) | | 1.96 (0.61,6.29) | | 0.87 (0.71,1.07) | | 1.28 (0.86,1.91) | 0.98 (0.65,1.49) | |
| Age and sex adjusted  HR (95% CI)* | 1.10 (0.98,1.22) | 1.40 (0.99,1.98) | 0.74 (0.34,1.59) | 1.11 (0.89,1.39) | 1.35 (1.01,1.80) | | | 1.47 (1.06,2.03) | | 1.60 (0.50,5.19) | | 0.85 (0.69,1.05) | | 1.08 (0.72,1.62) | 0.81 (0.54,1.24) | |
| **Hypertension** |  |  |  |  |  |  |  |  |  |  |  |  |  |  |  |  |
| Unadjusted  HR (95% CI) | 1.24 (1.14, 1.35) | 0.99 (0.72, 1.37) | 0.87 (0.49, 1.54) | 1.12 (0.95, 1.33) | 1.20 (0.98, 1.46) | | | 1.08 (0.85, 1.36) | | 1.40 (0.63, 3.10) | | 0.98 (0.82, 1.18) | | 1.83 (1.41, 2.37) | 1.41 (1.03, 1.93) | |
| Age and sex adjusted  HR (95% CI)* | 1.20 (1.1, 1.31) | 0.94 (0.67, 1.32) | 0.79 (0.44, 1.40) | 1.00 (0.84, 1.20) | 1.18 (0.96, 1.44) | | | 1.06 (0.84, 1.34) | | 0.85 (0.38, 1.89) | | 0.96 (0.79, 1.15) | | 1.44 (1.1, 1.88) | 1.01 (0.73, 1.39) | |
| **Dyslipidemia** |  |  |  |  |  | | |  | |  | |  | |  |  | |
| Unadjusted  HR (95% CI) | 0.76 (0.64, 0.92) | 0.52 (0.19, 1.38) | 2.59 (1.06, 6.31) | 0.78 (0.50, 1.23) | 0.74 (0.35, 1.57) | | | 0.59 (0.19, 1.82) | | 2.82 (0.69, 1.59) | | 0.93 (0.44, 1.96) | | 1.47 (0.76, 2.86) | 0.55 (0.39, 0.79) | |
| Age and sex adjusted  HR (95% CI)* | 0.77 (0.64 0.92) | 0.48 (0.18, 1.30) | 2.46 (1.01, 6.01) | 0.74 (0.47, 1.17) | 0.74 (0.35, 1.56) | | | 0.59 (0.19, 1.82) | | 2.86 (0.68, 12.09) | | 0.91 (0.44, 1.92) | | 1.29 (0.67, 2.51 | 0.53 (0.37, 0.76) | |
| **Myocardial infarction** |  |  |  |  |  |  |  |  |  |  |  |  |  |  |  |  |
| Unadjusted  HR (95% CI) | 1.84 (1.28, 2.63) | 0.05 (0.00, -) | 0.05 (0.00, -) | 1.39 (0.20, 9.84) | 3.43 (1.43, 8.27) | | | 0.55 (0.08, 3.88) | | 0.05 (0.00, -) | | 1.60 (0.40, 6.41) | | 0.05 (0.00, -) | 4.71 (1.51, 14.69) | |
| Age and sex adjusted  HR (95% CI)* | 1.72 (1.20,2.47) | 0.00 (0.00, -) | 0.00 (0.00, -) | 1.26 (0.18, 8.97) | 3.32 (1.38, 8.00) | | | 0.52 (0.07, 3.72) | | 0.00 (0.00, -) | | 1.52 (0.38, 6.08) | | 0.00 (0.00, -) | 2.80 (0.89, 8.80) | |
| **Heart failure** |  |  |  |  |  | | |  | |  | |  | |  |  | |
| Unadjusted  HR (95% CI) | 1.95 (1.68, 2.27) | 7.06 (4.28, 11.64) | 7.59 (2.81, 20.50) | 2.29 (1.58, 3.31) | 2.29 (1.51, 3.46) | | | 6.03 (3.55, 10.24) | | 15.24 (2.09, 110.96) | | 2.06 (1.29, 3.28) | | 4.10 (1.83, 9.18) | 1.36 (1.00, 1.85) | |
| Age and sex adjusted  HR (95% CI)* | 187 (1.60, 2.16) | 6.87 (4.14, 11.38) | 7.37 (2.73, 19.91) | 2.04 (1.41, 2.97) | 2.22 (1.46, 3.37) | | | 5.93 (3.48, 10.11) | | 4.45 (0.57, 34.71) | | 2.00 (1.25, 3.20) | | 2.78 (1.24, 6.24) | 1.06 (0.78, 1.45) | |
| **Atrial fibrillation** |  |  |  |  |  | | |  | |  | |  | |  |  | |
| Unadjusted  HR (95% CI) | 1.56 (1.25, 1.95) | 1.38 (0.19, 9.83) | 7.86 (1.95, 31.71) | 2.42 (1.34, 4.39) | 1.03 (0.57, 1.86) | | | 6.52 (3.36, 12.62) | | 0.05 (0, -) | | 1.57 (0.65, 3.77) | | 3.27 (1.46, 7.32) | 3.12 (1.00, 9.72) | |
| Age and sex adjusted  HR (95% CI)* | 1.47 (1.17, 1.84) | 1.24 (0.18, 8.88) | 7.22 (1.78, 29.21) | 2.14 (1.18, 3.88) | 0.98 (0.54, 1.79) | | | 6.30 (3.23, 12.30) | | 0 (0, -) | | 1.52 (0.63, 3.67) | | 2.37 (1.06, 5.32) | 2.11 (0.67, 6.61) | |
| **Stroke** |  |  |  |  |  | | |  | |  | |  | |  |  | |
| Unadjusted  HR (95% CI) | 1.20 (1.07, 1.35) | 1.09 (0.48, 2.43) | 1.55 (0.61, 3.90) | 1.43 (1.05, 1.95) | 1.46 (1.11, 1.91) | | | 1.16 (0.80, 1.67) | | 2.52 (0.79, 8.10) | | 1.13 (0.74, 1.72) | | 2.56 (1.53, 4.28) | 1.43 (1.01, 2.03) | |
| Age and sex adjusted  HR (95% CI)* | 1.15 (1.02, 1.29) | 1.04 (0.46, 2.35) | 1.35 (0.53, 3.45) | 1.26 (0.92, 1.72) | 1.43 (1.09, 1.88) | | | 1.14 (0.79, 1.64) | | 0.73 (0.22, 2.41) | | 1.09 (0.71, 1.67) | | 2.03 (1.21, 3.39) | 1.03 (0.72, 1.47) | |
